# Proposition of irrigation system for wetting the clay surface of tennis courts

**DOI:** 10.1371/journal.pone.0275571

**Published:** 2022-10-05

**Authors:** Carlos Henrique Ramalho Ferens, Carlos Augusto Brasileiro de Alencar, Giovanna Lyssa Lacerda Costa, Jean Carlos Coelho Pacheco, Lucas Maltoni Andrade, Roberto Filgueiras, Fernando França da Cunha

**Affiliations:** Department of Agricultural Engineering, Center of Agricultural Sciences, Federal University of Viçosa, Viçosa, MG, Brazil; Ningbo University, CHINA

## Abstract

The key to maintaining a clay court with quality and lastingly is through water applications, carried out periodically and through systems with high distribution uniformity, developed specifically for this purpose. The objective in this study was to evaluate the performance of a sprinkler irrigation system with hose and shower, traditionally used in clay tennis court, and propose another low-cost system that is operational and technically feasible, which is the irrigating bar. For each irrigation system, three evaluations were performed. At the beginning of each test, the pressures and flow rates of the emitters were measured, and the water distribution profile method was used to determine the distribution uniformity of the systems. Distribution efficiency was obtained through the Christiansen’s (U_C_), distribution (U_D_), absolute (U_A_), statistical (U_S_) and Hart’s (U_H_) uniformity coefficients, HSPA standard efficiency (U_HSPA_) and, coefficient of variation (CV). Subsequently, the application and irrigation efficiencies were calculated. It was found that the irrigation bar required lower operating pressure, as well as greater stability of pressure and flow in relation to the hose system. Water losses in the hose/shower system (22.0%) were higher than in the irrigation bar (0.6%). Regardless of the evaluated system, U_C_ (68.4% and 86.5%) and U_H_ (66.4% and 87.5%) values were similar and higher than those of the other coefficients (~51.8% and ~81.2%). The collected depths, applied by the hose/shower irrigation system, showed high spatial variability and, consequently, low values of uniformity, being classified as poor or unacceptable. The irrigating bar promoted higher values of uniformity coefficients, being classified as good. Irrigation efficiencies were 53.97 and 85.97% for hose/shower and irrigation bar systems, respectively. The hose/shower system has low performance in the irrigation of clay tennis courts. The irrigation bar system, for providing technical, operational, and economic benefits, and has the potential to be used in the irrigation of clay tennis courts.

## 1. Introduction

Tennis is played on a flat rectangular surface, usually of grass, clay or on hard ground. The dimensions of the court’s useful area are 23.77 and 10.97 m in length and width, respectively [1]. There is also additional space around the court, which is required for players to reach the ball when it passes the boundaries of the court [2]. Thus, the total length of the court is 36 m and the total width is 18 m, resulting in a total area of approximately 650 m^2^.

Clay has the greatest friction among all floors, which results in a slower style of play, because the ball, when bouncing, loses speed and rises higher [3]. In addition, due to the particles of brick dust, clay allows for a great slide of the players’ feet, which can be beneficial in some situations, although it can also cause slips in others. On this court, the points are longer and benefit players who prefer to play at the bottom of the court. These characteristics make the clay floor the most used in tennis courts throughout Brazil. Some famous tournaments played on this floor are Roland Garros and Monte Carlo, in addition to those that take place in Brazil, such as Brazil Open and Rio Open [4].

The brick powder of clay courts dries quickly due to climatic factors such as solar radiation, vapor pressure deficit, air temperature, wind speed etc [5, 6]. Thus, the court needs to receive constant and uniform hydration, otherwise cracks will be generated on its surface, enabling the growth of vegetation and reducing its quality and useful life. Irrigation also has the function of ensuring the softness of the floor, firmness of the soil and preventing the wind from lifting dust particles and causing discomfort to players and dirt in neighboring locations. When the clay court is not being used, the ideal is to sweep and irrigate it twice a day. On days of use, the need for irrigation is greater, but the amount of water should be enough so that the court is always in conditions for use in the next match. For this, the employee must respect various weather elements, that is, initial moisture, climatic factors of the day, moments of shade etc [4, 7].

Traditionally, the irrigation of clay courts in Brazil is performed through sprinkler systems using hoses with a shower device at its end [7]. An advantage of this system is the operationality of use and the low costs for its acquisition. However, this system has many disadvantages because the application is manual and does not follow any operating standard. The water jet and its direction is manually controlled by the operator. Thus, the overlap of jets and irrigation time in each subarea of the tennis court are defined by subjective criteria, which can cause uneven water distribution. In addition, the system needs to operate at high operating pressures for the jet to be fragmented. This results in a higher energy expenditure and reduces drop diameter. The reduction of drop diameter, associated with the great distances traveled by water, from the emitter to the target to be irrigated, intensifies the effects of climatic factors on water application [8–10]. Therefore, depending on the weather conditions, there may be greater water losses, reducing irrigation efficiency.

Considering that clubs and condominiums have more than one tennis court, that various irrigation events are performed per day and that the courts have large areas, the volume of water used in irrigations is expected to be large. Thus, any fraction of irrigation lost will also result large wastes of water and electricity. Considering that the vast majority of tennis courts are installed in the urban area, where the prices of water and electricity are higher, the economic damage can be even more significant.

In addition to economic problems, we should also consider the environmental problem, because good quality water is a finite resource and needs to be used rationally. Thus, it is of great importance that water users adopt systems with high irrigation efficiencies in order to reduce water use [10, 11]. There is no study in the literature proposing another system for wetting clay courts or evaluating the hose/shower system. In a recent study, Rodrigues and Afonso [12] even propose to use alternative ways of capturing water to wet tennis courts in Portugal. This corroborates the concern with water resources, however, evaluation and development of more efficient systems had not yet been studied. Thus, irrigation systems must be constantly developed or improved to meet these demands. In this process, universities and research companies are fundamental, and it is evident that studies that propose the development of more efficient irrigation systems are necessary and relevant.

To choose or replace an irrigation system with a more efficient one, evaluations must be carried out [9]. It is worth mentioning that the performance of irrigation systems should be quantified in the initial phase of planning and management and, in the field, as an operational criterion. Based on these, we believe that the hose/shower system for wetting clay courts has low water application and distribution efficiency, and that this performance can be improved through the development of a new system. In this way, we will be able to significantly reduce water consumption to moisten clay tennis courts. Thus, the objective of this study was to evaluate the performance of a sprinkler irrigation system with hose and shower, traditionally used on clay tennis courts, and to propose another low-cost system that is operational and technically feasible.

## 2. Material and methods

Two irrigation systems for a clay tennis court were evaluated. The first irrigation system evaluated was the one which uses a hose with a shower device at its end, here called the hose/shower system. This is the traditional way of irrigating clay tennis courts throughout Brazil. The second irrigation system was an adaptation of a system that consisted of a bar with seven emitters installed, here called an irrigating bar. These evaluations were carried out in Viçosa-MG, Brazil, between September and December 2020.

### 2.1. Experiment 1: Hose/shower system

**Fig 1:**
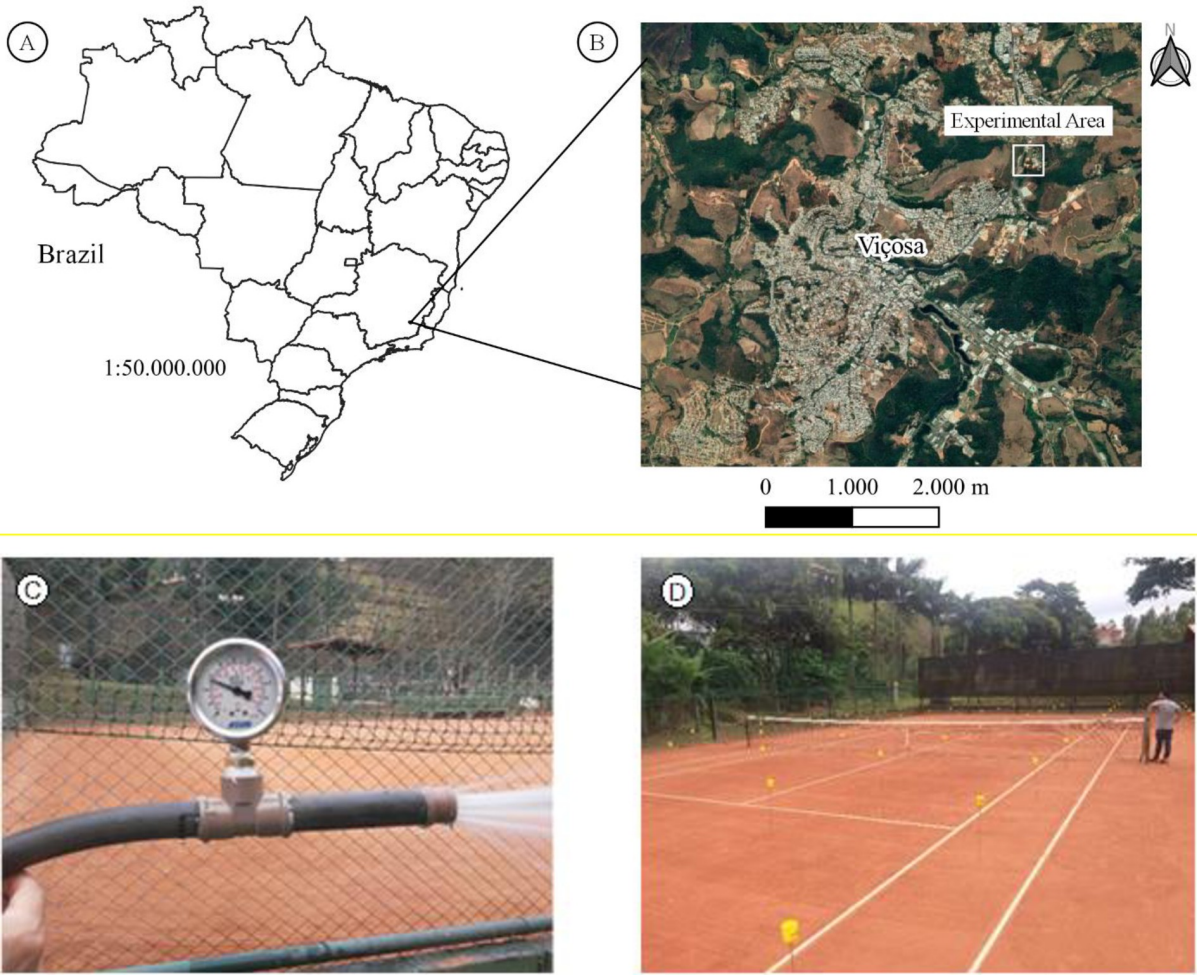
Locations of the experimental area in relation to Brazil (A), the Municipality of Viçosa-MG (B), detail of the hose with the shower-type emitter (C), and distribution of collectors on the tennis court (D). The figure was elaborated using the open source software QGIS [14]—using the basemap: Google Satellites (obtained through HCMGIS [15] QGIS plugin).

The study was conducted on the tennis court number 3 of *Clube Campestre de Viçosa* (Fig 1A and 1B), located in Viçosa-MG, whose geographic coordinates are: latitude 20°44’30.84” S, longitude 42°51’48.96” W and altitude of 703 m, in the *Zona da Mata* mesoregion. The local climate is Cwa, according to the Köppen-Geiger classification, that is, a humid subtropical climate with dry winter and hot summer [13]. The tennis court is 18 m wide and 36.1 m long, with total area of 650 m^2^. Tennis court clay has field capacity, wilting point, and apparent density of 0.2962 cm cm^-3^, 0.1698 cm cm^-3^, and 1.22 g cm^-3^, respectively. Above the clay, there is a layer of approximately 1 mm of tile dust.

The irrigation system used to irrigate the tennis court of *Clube Campestre de Viçosa*, and in most courts in Brazil, consists of PVC plastic hoses, similar to those used for garden irrigation, connected to a shower-type nozzle. The hose used in the experiment was ¾" in diameter (Fig 1C). The water jet and its direction are manually controlled by the operator. Thus, the uniformity of distribution and the water depth to be applied are also a function of the handling of the equipment. The operator uses subjective criteria such as a change in the color of the clay or the formation of a mirror of water on the surface of the court to complete the operation. However, as an advantage of this system is the ease of use and the low costs for its acquisition.

The water distribution profile method was used to determine the distribution uniformity of the system on the surface. This was carried out using the precipitation kit of the manufacturer Fabrimar^®^, which consists of aluminum rods and plastic water collectors and graduated cylinders. The water collectors were arranged throughout the tennis court and were installed at equidistant spacings of 3 m (Fig 1D). Thus, each collector represented a quadrangular area of 9 m^2^, following the Brazilian standard ABNT-NBR: 14244 [16]. Six columns and 12 rows of collectors were installed, resulting in a total of 72 collectors to represent the tennis court, sampling an area of 648 m^2^. The collectors were suspended by a rod at 70 cm height from the soil surface, following the methodology proposed by Merriam and Keller [17].

Three field tests were carried out under different climatic conditions during the period of September and November of 2020. At the beginning and end of each test, through three repetitions, the pressures and flow rates of the shower were measured. The pressures were obtained by means of a glycerin-filled pressure gauge and the flow rates were obtained by direct method. At the end of each test, the water depths in each collector were measured with a graduated cylinder. The collected volumes were converted to water depths (mm), by dividing the collected volume (L) by the collector area (m^2^).

### 2.2. Experiment 2: Irrigating bar system

The study was conducted at the Hydraulics Laboratory of the Agricultural Engineering Department (DEA) of the Federal University of Viçosa (UFV), located in the municipality of Viçosa-MG, whose geographic coordinates are: latitude 20°46’19.56” S, longitude 42°52’27.48” W and altitude of 667 m.

**Fig 2:**
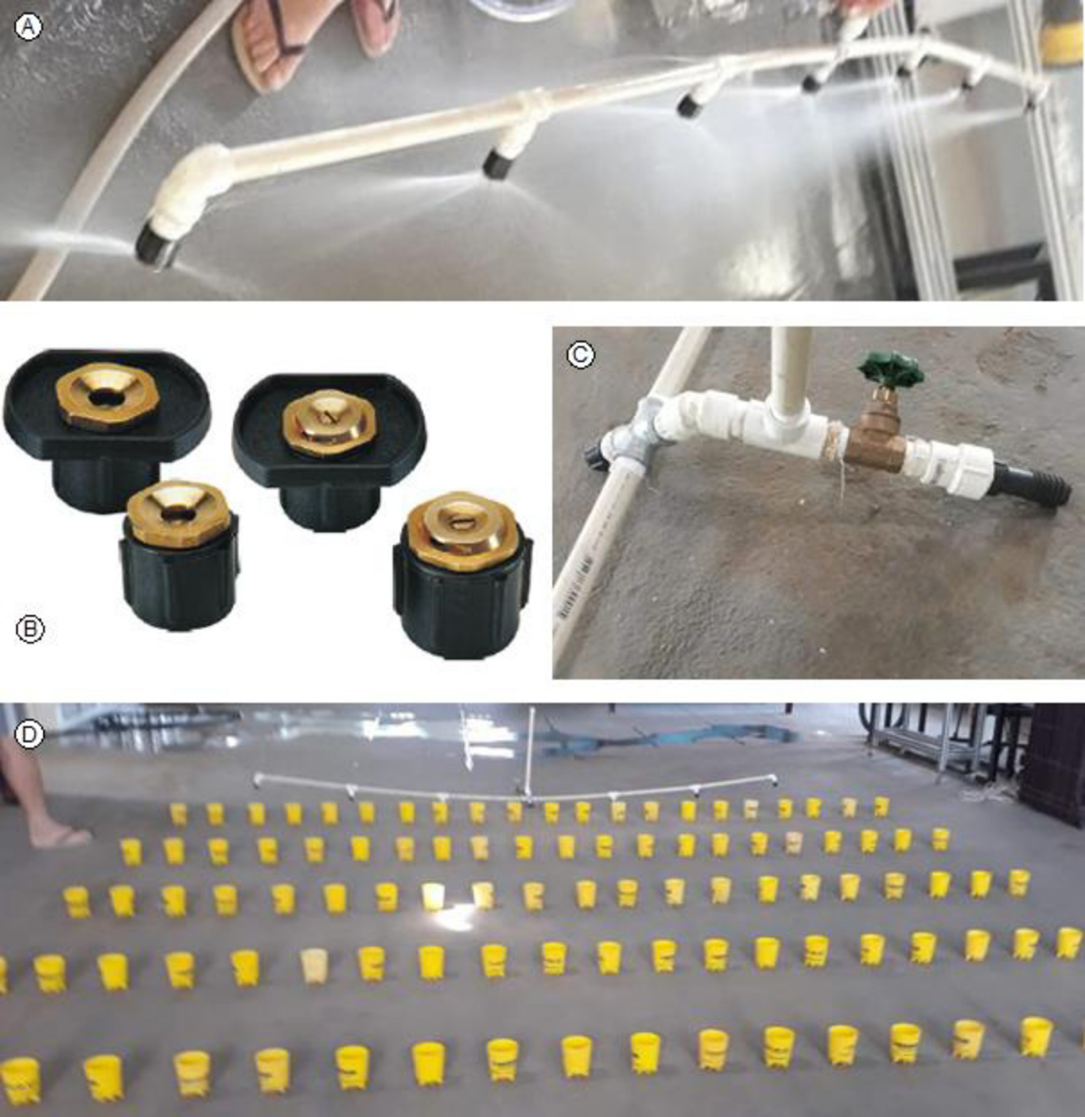
Irrigating bar (A), SempreVerde emitters from Fabrimar^®^ (B), base/support with constituent parts (C) and overview of the distribution of water collectors for system evaluation (D).

**Fig 3:**
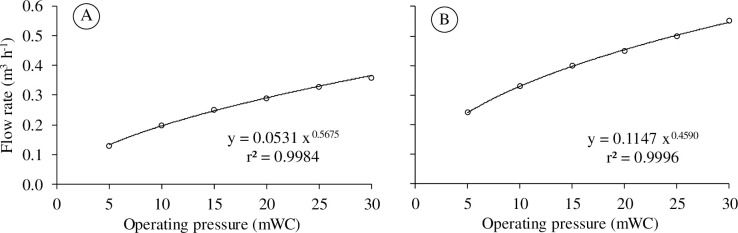
Flow rate versus operating pressure curve for SempreVerde emitters from Fabrimar^®^ with opening angles of (A) 90° and (B) 180.

In this test, an irrigation device was assembled using a PVC bar with 12 mm in diameter and 3 m in length (Fig 2A). On this bar, seven emitters (Fig 2B) of the SempreVerde model from the manufacturer Fabrimar^®^ were installed, spaced by 0.5 m. The two emitters installed at the end of the bar worked with an opening angle of 90° (SempreVerde 90) and the five emitters on the inside of the bar worked with an opening angle of 180° (SempreVerde 180). The operating pressure versus flow rate curves of the emitters are shown in Fig 3. In addition to the PVC bar and the emitters, the device also has a base/support (Fig 2C) consisting of materials that are presented in Table 1, which in total result in the final cost of R$314.10 (U$58.06 on 01/27/2021). This device was called irrigating bar.

**Table 1:** Constituent materials of the irrigating bar with their respective quantities and average cost for their acquisition.

Description	Quantity	Price (R$)	
		Unit	Total
SempreVerde 90° Emitter	2	19.50	39.00
SempreVerde 180° Emitter	5	19.50	97.50
½" Threaded PVC pipe	1	64.40	64.40
¾" Metal cross fitting	1	19.50	19.50
½" PVC nipple	7	1.95	13.65
½" Threaded 90° elbow	2	3.90	7.80
½" Treaded PVC tee	5	3.90	19.50
¾" Insert adapter	1	2.95	2.95
¾" Threaded ball valve	1	21.50	21.50
¾" Threaded PVC union	2	9.75	19.50
¾" Threaded cap	1	3.90	3.90
¾" Threaded 90° elbow	1	4.90	4.90
Total			314.10

* Values obtained at the local market.

The evaluation of the irrigating bar system was similar to the procedures already presented for the hose/shower system. In the evaluation of the irrigating bar, 5 rows and 21 columns of water collectors were installed (Fig 2D). The water collectors were arranged with spacings of 0.2 m in the transverse direction in relation to the bar (in the same row) and 0.8 m in the longitudinal direction (in the same column). The area evaluated had dimensions of 4.2 m (row) by 4.0 m (column), resulting in an area of 16.8 m^2^. Three tests were performed in December 2020.

### 2.3. Evaluation of the performance of irrigation systems

After obtaining the data in the evaluations, weighted interpolations (weight = 2) were performed by inverse distance weighting (IDW) to have an idea of the spatial distribution of the irrigation depths for the different systems tested. In the simulations, the data of collected depth were extrapolated to the spacing of 1 m.

Subsequently, the uniformities of water distribution were calculated using Christiansen’s uniformity coefficient [18], distribution uniformity coefficient [19], absolute uniformity coefficient [20], statistical uniformity coefficient [21], Hart’s uniformity coefficient [22], HSPA standard efficiency [22] and the coefficient of variation by Eqs 1, 2, 3, 4, 5, 6 and 7, respectively.

$$U_{C}=100\;[1-\frac{\sum_{i=1}^{n}|X_{i}-\bar{X}|}{n\;\bar{X}}]$$ (1)

$$U_{D}=100\;\frac{X_{25\%}}{\bar{X}}$$ (2)

$$U_{A}=50\;\left[\frac{X_{25\%}}{\bar{X}}+\frac{\bar{X}}{X_{12.5\%}}\right]$$ (3)

$$U_{S}=100\;\left[1-\frac{S}{\bar{X}}\right]$$ (4)

$$U_{H}=100\;\left\{1-\sqrt{\frac{2}{\pi }}\;\left(\frac{S}{\bar{X}}\right)\right\}$$ (5)

$$U_{HSPA}=100\;\left(1-1.27\;\frac{S}{\bar{X}}\right)$$ (6)

$$CV=\frac{100}{\bar{X}}\sqrt{\frac{\sum {\left(X_{i}-\bar{X}\right)}^{2}}{\left(n-1\right)}}$$ (7)

where U_C_ is Christiansen’s uniformity coefficient, in %; Xi is the precipitation observed in the collectors, in mm; $\bar{X}$ is the average precipitation, in mm; n is the number of collectors; U_D_ is the distribution uniformity coefficient, in %; ${\bar{X}}_{25\%}$ is the average of 25% of the total collectors, with the lowest precipitations, in mm; U_A_ is the absolute uniformity coefficient, in %; X_12.5%_ is the average of 12.5% of the total collectors, with the highest precipitations, in mm; U_S_ is the statistical uniformity coefficient, in %; S is the standard deviation of precipitation data, in mm; U_H_ is Hart’s uniformity coefficient, in %; U_HSPA_ is the HSPA standard efficiency, in %; and CV is the coefficient of variation, in %.

The uniformity coefficients were interpreted by following a proposal adapted from ABNT [16] and Mantovani [23], presented in Table 2.

**Table 2:** Classification of uniformity coefficient values for sprinkling systems.

Classification	U_C_ (%)	U_D_ (%)	U_S_ (%)	U_H_ (%)	U_HSPA_ (%)	CV (%)
Excellent	> 90	> 84	> 90	> 90	> 90	< 5
Good	80–90	68–84	80–90	80–90	80–90	5–10
Moderate	70–80	52–68	70–80	70–80	70–80	10–20
Poor	60–70	36–52	60–70	60–70	60–70	20–30
Unacceptable	< 60	< 36	< 60	< 60	< 60	> 30

Adapted from ABNT [16] and Mantovani [23].

To calculate the application efficiency (E_A_), first the evaporation and drift losses (EDL) were calculated using Eq 8. Through EDL, E_A_ and irrigation efficiency (Ei) were calculated using Eqs 9 and 10, respectively.

$$EDL=100\;\frac{Wa-Wc}{Wa}$$ (8)

$$E_{A}=100-EDL$$ (9)

$$Ei=U_{C}\;E_{A}$$ (10)

where EDL corresponds to evaporation and drift losses, in %; Wa is applied water depth, in mm; Wc is the collected water depth, in mm; E_A_ is the application efficiency, in %; Ei is the irrigation efficiency, in %; U_C_ is Christiansen’s uniformity coefficient, in decimal.

E_A_ was classified according to the methodology of Bralts [24], being ideal when E_A_ is greater than 95%, acceptable when E_A_ is between 80 and 95% and unacceptable when E_A_ is lower than 80%.

## 3. Results and discussion

The data referring to the evaluations of the different irrigation systems for tennis courts are presented in Table 3. The pressure and flow rate values remained constant during the evaluation period and between the evaluations, as observed in their respective standard deviations. This also suggests a low instability of the electrical grid and good quality of the water used in irrigations, since it was treated by the municipality’s supply company and, thus, did not contain suspended solids that could clog any component of the system.

**Table 3:** Means followed by standard deviation of the parameters obtained during evaluations of irrigation systems for tennis courts.

Parameters	Irrigation System			
	Hose/Shower		Irrigating bar	
Flow rate (m^3^ h^-1^)*	2.870	± 0.010	1.492	± 0.016
Pressure (mWC)	25.100	± 0.100	5.267	± 0.110
Application intensity (mm h^-1^)**	4.415	± 0.015	2.295	± 0.024
Application time (min)**	11.787	± 0.961	20.298	± 0.632
Average depth applied (mm)	0.867	± 0.073	0.776	± 0.024
Average depth collected (mm)	0.677	± 0.061	0.771	± 0.016
Average wind speed (m s^-1^)	0.867	± 0.351	0.000	± 0.000

* For the irrigating bar, the sum of the flow rates of the seven emitters installed was considered.

** Considering the entire tennis court (650 m^2^).

The hose irrigation system operated with higher operating pressure (Table 3) because the shower device required the water to be sprinkled, that is, the water jet was fractionated in the nozzle and by the contact with the atmosphere, simulating a rain. On the other hand, the lower operating pressure required by the irrigating bar system has the advantage of lower electricity consumption. The irrigating bar, even with seven emitters, had lower flow rate and water application intensity compared to the hose irrigation system with the shower device connected at the end of the line. Thus, using the same amount of equipment and labor, the hose/shower system will require a shorter time to wet the tennis court for the application of the same gross irrigation depth.

Table 3 also shows that the difference between the applied and collected irrigation depths was higher for the hose/shower irrigation system (22.0%) when compared to the irrigating bar system (0.6%). Thus, it is possible to infer that the water losses in the hose/shower system were higher than in the irrigating bar. This can be attributed to the lower values of operating pressure and wind speed that occurred in the evaluation of the irrigating bar system.

In irrigation systems in general, part of the depth applied by the emitters does not reach the soil surface and/or target. This portion of water represents the losses by evaporation and drift (EDL), which in turn are expressed as the percentage of the gross volume applied lost in a given irrigation event [25]. Evaporation losses are higher in places with lower values of relative humidity and higher values of wind speed and air temperature. Evaporation losses are also higher in systems that apply water with smaller drop diameter, which in turn is a function of the emitter characteristics and operating pressure. Drift losses depend mainly on wind speed, drop diameter and distance traveled by water until reaching the target to be irrigated [9, 10, 26]. Thus, it can be noted in the hose/shower irrigation system that, in addition to the higher values of operating pressure and wind speed, another characteristic that contributed to the greatest losses was the fact that water was applied from a distance. In the hose/shower system the distance between target and emitter can reach up to 6 m and, in the irrigating bar system, the target is approximately 30 cm away from the emitter.

**Fig 4:**
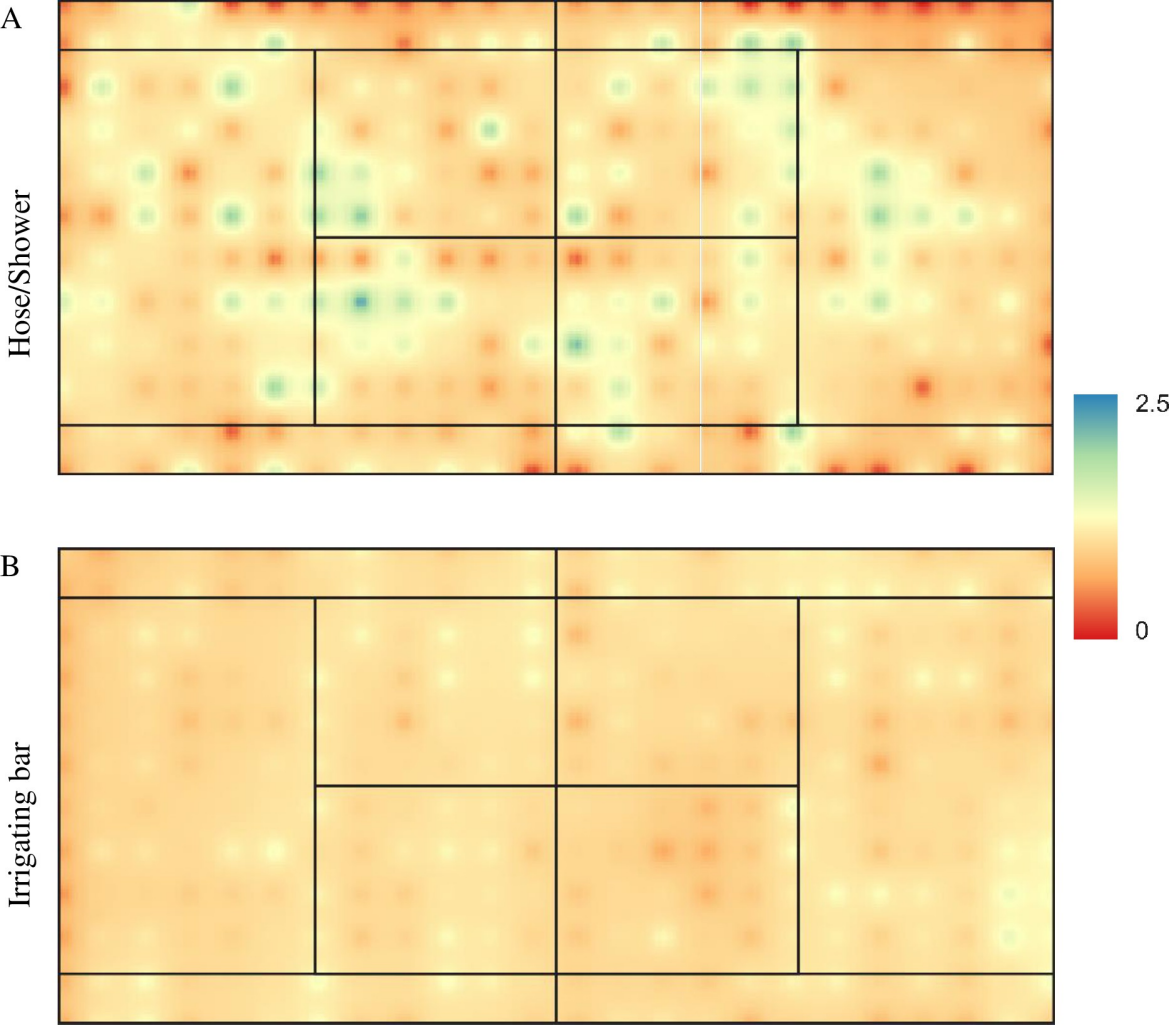
Spatial distribution of water depths collected by irrigation systems by (A) hose connected to a shower-type emitter and (B) irrigating bar, both for irrigation of clay tennis courts.

The spatial distributions of the collected depths, after water applications by the hose system equipped with a shower and the irrigating bar system, are presented in Fig 4. It can be observed that the figure was created simulating an application of an average irrigation depth of 1 mm. This strategy of using the same average depth was chosen to establish a better visual comparison between irrigation systems. There was a greater non-uniformity of distribution of irrigation depths in the hose/shower system. This also occurred due to the factors already mentioned, such as higher operating pressure, greater distance between the emitter and target, and higher wind speed. In addition, it must also be considered that the application of water by the hose/shower method is manual and at specific points, hence not following any application pattern. Thus, the overlap of the jets and the irrigation time in each subarea of the tennis court are defined by subjective criteria of the operator.

It should be noted that clay tennis courts have low infiltration capacity. This occurs because they are built with compacted layers of approximately 5 cm. Thus, even irrigations with low intensity of water application are capable of causing puddles. Thus, at the beginning of irrigation, the flow process begins, which is noted by the characteristic mirroring aspect of the court’s surface. The operator, to minimize the non-uniformity, aims the water jet at subareas that show a matte brown color and, thus, the entire tennis court is irrigated. Given the above, it is already imagined that the water distribution uniformity will not be adequate, and this can be confirmed in Fig 4A.

Table 4 shows the water distribution uniformity coefficients, by different methodologies, calculated for the evaluations performed in the different irrigation systems. As already verified in Fig 4A, the hose/shower irrigation system had low values of uniformity coefficients, being classified as poor or unacceptable. Thus, the results indicate that the hose/shower system should not be recommended for irrigation of clay tennis courts, considering the uniformity of distribution and the saving of water and electricity. However, other studies should be conducted, since the results found here are only for the application of water on the tennis court’s surface, and it is believed that after water is applied to this surface, it is redistributed in the first layers of clay, as suggested by Araújo et al. [9] and Rocha et al. [27].

**Table 4:** Values of uniformity coefficients and efficiencies, with their respective deviations and classifications, for different irrigation systems for tennis courts.

Parameters	Irrigation system			
	Hose/Shower		Irrigating bar	
Christiansen’s Uniformity Coefficient	68.43	± 4.25	86.49	± 1.48
(U_C_ in %)	Poor		Good	
Distribution Uniformity Coefficient	50.43	± 7.69	78.87	± 1.27
(U_D_ in %)	Poor		Good	
Statistical Uniformity Coefficient	57.82	± 5.79	84.28	± 1.19
(U_S_ in %)	Unacceptable		Good	
Hart’s Uniformity Coefficient	66.35	± 4.62	87.46	± 0.95
(U_H_ in %)	Poor		Good	
HSPA standard efficiency	47.28	± 7.24	80.35	± 1.48
(U_HSPA_ in %)	Unacceptable		Good	
Coefficient of Variation	42.17	± 5.79	15.72	± 1.19
(CV in %)	Unacceptable		Moderate	
Application efficiency (E_A_ in %)	78.44	± 10.76	99.40	± 1.04
	Unacceptable		Ideal	
Irrigation efficiency (Ei in %)	53.97	± 10.85	85.97	± 1.77

Given the low uniformity of water distribution by the hose/shower method, the irrigating bar system is proposed in replacement. Fig 4B indicates that the water distribution by the irrigating bar shows less spatial variation of the collected depths, and its best uniformity is confirmed by the coefficients presented in Table 4. The irrigation system by irrigating bar, in comparison to the hose/shower system, showed gains of 18, 28, 26, 21 and 33% for U_C_, U_D_, U_S_, U_H_ and U_HSPA_, respectively. The coefficient of variation (CV) of the depths collected in the irrigating bar system was 26% lower than that obtained with the other system. These uniformity gains led to better classifications of distribution uniformity coefficients, with performances mostly classified as good.

Table 4 also shows that the standard deviation values in the evaluations performed in the irrigation bar system were lower than those for the hose/shower system. This shows that, in addition to having greater uniformity of distribution, the irrigating bar promotes applications with the same performances in the different irrigation events. It should also be considered that the averages of the uniformity coefficients calculated for the irrigating bar system are higher, and this would contribute to the standard deviations being also higher.

As commented for the hose/shower system, in the irrigation bar system the irrigation performance will also be influenced by the operator. In the longitudinal direction to the irrigating bar, the irrigation performance will depend on the uniformity of the operator’s speed while moving the irrigating bar. Thus, the operator’s speed variation will be directly proportional to the performance of the irrigation application. On the other hand, in the transverse direction to the irrigating bar, the irrigation performance will depend on the uniformity of the flow rate and water distribution by the emitters installed on the bar. Hence, it can be noted that the operator will cause less influence on the irrigating bar system than on the hose/shower system.

Although there is a numerical difference, the uniformity coefficients showed similar behaviors in the evaluation of the different irrigation systems (Table 4). It is also observed in this table that the values of U_C_ and U_H_ were similar. This was already expected, since Hart [22], developer of U_H_, reports that, when the water depth applied by sprinklers has normal distribution, U_C_ is equal to U_H_. The values of U_C_ and U_H_ were higher than those of the other coefficients evaluated, as observed in the literature [9–11, 28]. Filgueiras et al. [11], working with these same uniformity coefficients to evaluate a conventional sprinkler irrigation system, found that U_C_ had the highest values, followed by U_H_, U_S_, U_D_ and U_HSPA_. U_D_ is more restrictive and will generally be lower than U_C_, since only 25% of the collectors that received the smallest amount of water are considered in the calculation of U_D_. Keller and Bliesner [8] also stated this and added that U_D_ may be related to U_C_ by the expression: U_D_ = 100–1.59 (100—U_C_). When U_D_ was calculated with this equation, there was an average underestimation of only 0.84% compared to the U_D_ value obtained through the depths collected in the present study.

The efficiencies of application (E_A_) and irrigation (Ei) were higher in the evaluations for the irrigation bar system (Table 4). In this system, E_A_ received an “ideal” classification and was 21% higher than that of the hose/shower system, which received an “unacceptable” classification. One of the reasons for this great difference was the weather conditions because, while in the irrigation bar system the evaluation was performed inside a shed, the evaluations of the hose/shower system were performed in the open air, on the tennis court itself, where higher wind speeds occurred. Thus, the same comments made for EDL also serve for Ei, and this parameter is obtained through EDL in %. Despite that, considering the same weather condition, the irrigating bar is expected to have higher values of E_A_. In this system, the application of water is localized, with distances around 30 cm between emitter and target. Thus, climate conditions will have less influence on EDL.

Ei was calculated by multiplying E_A_ by the distribution efficiency, where U_C_ was used. In this way, an adequately irrigated area of 80% is expected [8]. In the evaluation of the irrigating bar system, Ei was 32% higher than in the hose/shower system. In practical terms, for the application of a net depth of 0.5 mm, the irrigating bar and hose/shower systems will need to apply gross depths of 0.58 and 0.93 mm, respectively. Considering the entire tennis court (650 m^2^), the water volumes applied in irrigation would be 378 L (irrigating bar) and 602 L (hose/shower), so the irrigating bar system would save 224 L of water, in addition to electricity. Disregarding E_A_ in the calculation of Ei, since it was higher in the hose/shower system due to the different conditions in the study, the water saving would be 99 L of water. This saving is still significant because it should be considered that the clay tennis court is irrigated between the sets of the game, and there may be several irrigation events in a single day.

In view of all the above, it can be noted that the irrigating bar has technical potential to be used in the irrigation of clay tennis courts and, certainly, on grass tennis courts. This system also has the advantage of being low cost, because the replacement of the shower with the irrigating bar would cost R$ 314.10, value obtained on January 27, 2021, when the price of the dollar was U$ 5.41. However, *in loco* studies should be carried out in order to evaluate the performance of the irrigation system under different climate conditions and irrigation equipment configurations. Furthermore, as the hose/shower system is influenced by humans, further studies with different operators should be carried out to confirm our results.

Regarding the developed equipment, the authors believe that research using different emitters and operating pressures is also necessary. These new settings will affect flow rate, application intensity, drop diameter, etc., and can also contribute to improving irrigation efficiency by the irrigating bar.

## 4. Conclusions

The hose/shower system should not be recommended for irrigation of clay tennis courts, considering the distribution uniformity and savings of water and electricity.

The irrigating bar, for providing technical, operational and economic benefits, has the potential to be used in the irrigation of clay tennis courts.

## Supporting information

S1 File Evaluation data of hose/shower systems and irrigation bar for wetting the clay surface of tennis courts. (XLSX)
